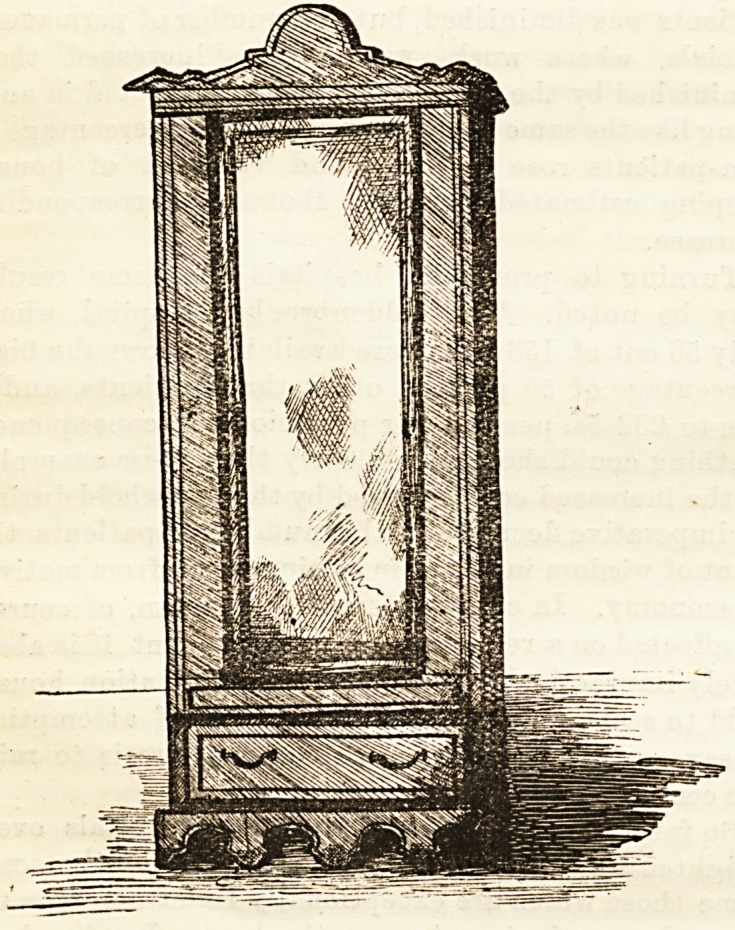# Practical Departments

**Published:** 1895-10-19

**Authors:** 


### PRACTICAL DEPARTMENTS.

### FURNITURE AND FITTINGS FOR NURSES' HOMES.—II.

Viewed from a modern standpoint, as a whole, the great hospitals in London cannot be said to be well off in the quarters provided for their nursing staffs, that is to say when compared with such buildings as the new Nursing Home of the Edinburgh Royal Infirmary, and others of quite recent erection. In most cases the best has had to be made of quite insufficient material, and with the enormously increased staffs needed for the nursing requirements of to-day as compared with those of thirty or forty years ago, the authorities of the older hospitals have been hard put to it to find the necessary space for their accommodation. One of the most recent and best homes for nurses in London is that attached to the London Hospital, which was first opened in 188 , and well fulfils all hygienic requirements, besides being fitted with every necessary comfort and convenience. Each nurse has her sepaarte bedroom, and most cosy little rooms they are with their polished floors and strip of carpet, hanging cupboard with long glass, similar to the accompanying drawing, chest of drawers, washstand, table and chair, and spring-mattressed bed. The walls are distempered, and a good plan has been followed in the addition of a substantial beading at a convenient height for picture-hanging. Naturally, in rooms constantly changing ownership the knocking of nails into the wall at the sweet will of each occupant cannot be allowed, while it is hard upon nurses not to be able to collect their favourite photographs, &c., around them. The "London" plan settles the point at small cost. With tiny bed-rooms the question of ventilation is all important, and the best means of obtaining a sufficiency of fresh air with a minimum of draught is undoubtedly by a simple plan of having the lower sill of a sash window made a few inches higher than is necessary merely to meet the upper frame, allowing the sash to be raised a corresponding degree, the air thus entering the room in an upward direction between the sashes. A window open at the top on a cold night in a small room will make the atmosphere sometimes unpleasantly chilly, but the above method greatly modifies this. Throughout the Middlesex Hospital Institute for Trained Nurses, in Cleveland Street, this plan has been followed by the architect. There should, of course, be some form of ventilator over the door to admit during the day thorough cross ventilation.

An essential for a sitting-room devoted to the use of those whose work keeps them for many hours together on their feet is the provision of very easy chairs and sofas to give tired limbs free play. The wide and capacious ones covered with American cloth in the nurses' sitting-room at the London Hospital Home are good of their kind, and the room is altogether particularly comfortable looking. Nowadays wicker chairs well cushioned are popular, and nothing makes a more cosy lounge if discretion be exercised in the choosing, and the cushions are the right size and shape. The nurses' day-room at the Westminster Hospital, just re-done, and the sitting-room in the Nurses' Home of that hospital are exceedingly pretty home-like rooms, and both are well supplied with a variety of comfortable seats. When the sitting-room is large enough a screen or two of bamboo or cretonne add much to comfort at a trifling expense. It is somewhat curious and instructive to observe the change which has come about in late years in the relative standards of necessary comforts and luxuries. In comparing the simple home-like dining-hall and sitting-room of the Nightingale Home at St. Thomas's Hospital with the elaborate furnished sitting-room of the Edinburgh Royal Infirmary Nursing Home, which has all the appearance of a very handsome drawing-room, it may well be wondered whither the next development will lead, and if this present increase in what cannot but be called luxury be wise or necessary.

A very important feature is naturally the library, for without a supply of really good books, novels, and light reading as well as more standard literature, the "home" sitting-room cannot be considered completely furnished. The establishment of this can be so well helped forward by the nurses themselves with the exercise of a little energetic reminding to the friends of each, and responses should be found readily forthcoming. With a good library a bookcase with glass doors and lock and key is needed, for among many readers some system must perforce be observed if the books are to be kept in proper order and repair.

At St. Thomas's Hospital, as at King's, St. George's, and elsewhere, the nurses' rooms are placed at the top of the hospital itself. At St. Thomas's cubicles were originally arranged, but practical experience having universally proved these altogether undesirable in this case, they have been converted into separate rooms by carrying up the partition to the ceiling. Each cubicle had its window, and each door has its ventilator, therefore ventilation is well cared for. Besides the large general sitting-room, the St. Thomas's nurses have also a small sitting-room on their floor in each block, a pleasant arrangement for those who wish for some quieter place for retirement than the one shared by the whole staff of nurses. The newest addition in the way of bed-rooms has been the fitting up and adapting of a portion of the "apothecary's" house for the "theatre" sisters and nurses. These have been very completely and cosily furnished. The wardrobes have long mirrors, and are well made of light polished wood, and the combination dressing-table and chest of drawers, &c., are all good. More room is here to be made for the extra nurses required in consequence of the opening of two of the now closed wards.

## Figures and Tables

**Figure f1:**